# “They're Going to Zoom It”: A Qualitative Investigation of Impacts and Coping Strategies During the COVID-19 Pandemic Among Older Adults

**DOI:** 10.3389/fpubh.2021.679976

**Published:** 2021-05-19

**Authors:** Mikael Anne Greenwood-Hickman, Jacklyn Dahlquist, Julie Cooper, Erika Holden, Jennifer B. McClure, Kayne D. Mettert, Stephen R. Perry, Dori E. Rosenberg

**Affiliations:** Kaiser Permanente Washington Health Research Institute, Seattle, WA, United States

**Keywords:** aging, social isolation, stress, physical activity, sedentary behavior

## Abstract

**Introduction:** Older adults, who already have higher levels of social isolation, loneliness, and sedentary behavior, are particularly susceptible to negative impacts from social distancing mandates meant to control the spread of COVID-19. We sought to explore the physical, mental, and social health impacts of the pandemic on older adults and their coping techniques.

**Materials and Methods:** We conducted 25 semi-structured interviews with a sub-sample of participants in an ongoing sedentary behavior reduction intervention. Interviews were recorded and transcribed, and iterative coding was used to extract key themes.

**Results:** Most participants reported an increase in sedentary behavior due to limitations on leaving their home and increased free time to pursue seated hobbies (e.g., reading, knitting, tv). However, many participants also reported increased levels of intentional physical activity and exercise, particularly outdoors or online. Participants also reported high levels of stress and a large decrease in in-person social connection. Virtual connection with others through phone and video was commonly used to stay connected with friends and family, engage in community groups and activities, and cope with stress and social isolation. Maintenance of a positive attitude and perspective gained from past hardships was also an important coping strategy for many participants.

**Discussion:** The COVID-19 pandemic and associated social distancing measures have impacted older adults' perceived levels of activity, stress, and social isolation, but many leveraged technology and prior life experiences to cope. These themes could inform future interventions for older adults dealing with chronic stress and isolation.

## Introduction

The COVID-19 pandemic has impacted people across the globe, prompting the implementation of public health restrictions at the federal, state, and local level in an attempt to slow the spread of the virus and resulting disease. Older adults aged 65 or over with chronic conditions are at the highest risk for contracting and dying from a severe case of COVID-19, in addition to being at an increased risk for social isolation, loneliness, and high levels of sedentary behavior (1–5). In the United States, public health mitigation measures varied by state and began to take effect as early as March 2020. Washington state, which reported the first US case of COVID-19 (6), was one of the earliest states to announce social distancing and stay at home mandates in early March 2020. After more than 9 months of mandatory public health restrictions, such as physical distancing and public space closures, older adults are at an even higher risk for suffering negative social, mental, and physical impacts from these mandates (3, 7).

The short-term and long-term impacts of these restrictions on the health and well-being of older adults with chronic conditions is unclear. Early studies suggest that older adults are engaging in less physical activity and are more sedentary than before the pandemic, and that the biggest challenges presented by the pandemic are social constraints and activity restrictions (8, 9). In addition, initial studies suggest that social and emotional loneliness, anxiety, depression, and insomnia have increased for older adults (10, 11), and a recent mixed-methods analysis in the US identified themes of stress and loneliness as top concerns for older adults during the pandemic (12). While emerging evidence suggests that older adult populations have been negatively affected by the pandemic and resulting public health measures, the full extent of these impacts as well as the mechanisms used to cope with those changes have not yet been sufficiently investigated. Motivated during the early months of the pandemic when very little was known, our objective was to understand directly from older adult narratives how these events have broadly affected their mental, social, and physical health and to characterize the ways they were coping with these challenges. This information may aid development of future public health campaigns and interventions to better support older adult populations during this or future periods of prolonged social distancing or isolation.

## Materials and Methods

### Setting

The study was conducted at Kaiser Permanente Washington Health Research Institute located in Seattle, WA. Effective early March, 2020, Washington State began implementing state-mandated restrictions and business closures, and encouraging residents to stay at home, limit social gatherings, and maintain physical distance when in public spaces (13). All research activities were reviewed and approved by the Kaiser Permanente Washington Institutional Review Board. Interview participants were invited from the Healthy Aging Resources to Thrive (HART) trial, which began in February 2019 and is ongoing. Additional detail on the parent trial can be found at ClinicalTrials.gov (NCT03739762). All interviews were conducted between June and August of 2020.

### Brief Overview of the Parent Trial

HART is a randomized controlled trial that aims to reduce sitting time in older adults with a body mass index of 30 kg/m^2^ or above. Primary outcomes of interest are thigh-worn accelerometer-based sedentary behavior metrics and blood pressure measured at 6-months. Participants (*N* = 284; recruitment ongoing) are recruited from Kaiser Permanente Washington membership panels in King County, WA and are considered eligible if they are between age 60–89; have a body mass index between 30 and 50 kg/m^2^; self-report 6 or more hours of daily sitting time; are able to stand from a seated position without assistance; are able to walk one block; are fluent in English; have continuous enrollment within KPWA in the prior 12 months; and do not have indications in their medical record of long-term nursing care, palliative care, hospice care or a cancer diagnosis, deafness/significant hearing loss diagnosis, dementia, or a serious mental health disorder in the prior 24 months. Eligible and consenting participants who successfully complete all baseline study measurements are then randomized to receive a sitting reduction intervention (termed I-STAND) or a healthy living focused attention control condition. The I-STAND intervention is built on our team's prior work (14, 15) and includes 10 sessions with a health coach, which use motivational interviewing techniques and incremental goal setting to build awareness of and develop reminder strategies to sit less throughout the day. I-STAND participants also receive a wrist-worn prompting device and table-top standing desk as intervention tools. The healthy living control condition also includes 10 sessions with a health coach to set goals around various self-selected topics related topics of healthy aging (e.g., sleep, diet, stress reduction) Participants receive their assigned intervention for 6 months, at which point primary outcome measures are collected. After 6 months, those receiving the I-STAND condition are re-randomized to receive five booster health coaching sessions by phone over the subsequent 6 months or no further coaching contact; healthy living participants receive no further coaching contact. All participants are followed for an additional 6 months and have a final measurement assessment visit at 12 months to collect final study outcomes data. Participants receive a small cash incentive for completing each study measurement activity, including a bonus for completing all 4 measurement visits. No incentives are provided for intervention coaching visits; nor were additional incentives offered for those who participated in in-depth interviews.

### Qualitative Data Collection Procedures

HART participants who had recently completed their 6- or 12-month study measurement visit, which signal the end of active intervention and end of study follow-up, respectively, were considered eligible to participate in the qualitative interviews. Participants meeting these criteria were invited to participate and preference was given to those who had more recently completed a study visit in order to maximize participants' ability to recall details of study activities for intervention-related interview questions (not presented here). To maximize diversity, we also oversampled eligible participants of color and those with self-reported high blood pressure or diabetes at baseline. Eligible participants were contacted by phone or email and invited to participate in a 30–45 min telephone interview. Invitations continued until *N* = 25 participants, the minimum sample size to reach saturation recommended in the qualitative literature (16), had been interviewed. Those who agreed to participate and be audio-recorded, were orally consented *via* phone at the beginning of the interview phone call.

To reduce response bias, interviews were conducted by two team members who had no prior contact with the interview participants during the study. The interviewers (M.A.G.H. and J.D.) followed a semi-structured interview guide with open-ended questions and follow-up prompts. Interview questions focused on participants' experiences with the ongoing COVID-19 pandemic and included questions such as: “What impacts did you notice while social distancing measures were actively in place for our community?”; “Did you notice any specific impacts to your: mental health, physical activity levels, sitting time each day, diet or the foods you chose to eat each day, sleep patterns, or overall quality of life?”; “What has been helping you cope with the pandemic and related social distancing measures?”; “What impacts did the pandemic have on your overall engagement with the HART study, such as your ability to check in with your coach, meet goals, and make progress on your healthy behaviors?”; “Are there changes to your life that you think may not return to ‘normal' for a longer time?”. Interviews averaged 30 min (range = 15–54 min). Each call was audio-recorded and then transcribed for analysis. Participants were compensated for participation in the parent trial, but not compensated for completing the qualitative interview.

### Data Analysis

Each interview transcript was independently coded by at least two members of a three-person coding team. Transcripts were divided among two primary coders who collectively coded all transcripts and were the same individuals who conducted the in-depth interviews (M.A.G.H. and J.D.). M.A.G.H.'s background is in anthropology and public health and J.D.'s background is in psychology and public health. Both received qualitative methods training with supervision from investigators experienced in qualitative methods. They also had training in motivational interviewing from the lead researcher (D.E.R.). The primary coders were assisted by D.E.R., who has a background in clinical psychology and public health, including training in qualitative methods. Coding was performed using an inductive thematic approach (17) from the interview transcripts. Initially, a common code list was established by the primary coders based on their experience conducting and reading the interview transcripts several times. Repeated rounds of coding occurred over the course of several months and the code list and code book were refined during each round. Saturation was reached when no new codes were generated after a final review of the transcripts. Once a final code list was established and defined, all transcripts were coded independently for a final time (final codebook available in Appendix 1). Any differences were resolved through group consensus. Coding and final analysis was assisted by Atlas.ti 8.4 software (SAGE Publications, Thousand Oaks, CA). Output for each code from Atlas.ti was reviewed to identify key themes related to COVID-19 impacts and coping strategies reported by the study participants. Only codes with four or more participant quotations were included in the final results. Direct quotes from participants were selected to illustrate themes.

## Results

A total of *N* = 25 participants from the greater Seattle, WA area met study timepoint eligibility criteria and agreed to a one-time phone interview, and saturation was reached. Of the HART participants contacted, only one participant refused an interview due to scheduling conflicts. Table 1 describes characteristics of the interview participants. Participants were predominantly female (64%), white (88%), college educated (67%), and retired or working part-time (62%). Table 2 includes a summary of key themes and examples from participants related to COVID-19 pandemic impacts and coping strategies. Additional supporting quotations can be found in Appendix 2.

**Table 1:** Baseline demographic characteristics of interview participants.

	***N* Mean**	**% Range**
**Study arm**		
Intervention	16	64%
Booster^*^	11	44%
Attention control	9	36%
**Study time point**		
6-month	16	64%
12-month	9	36%
**Age**	68	60–77
**Gender**		
Female	16	64%
Male	8	32%
Non-binary	1	4%
**Race**		
White	22	88%
BIPOC	3	12%
Marital status^**^		
Married, living as married	14	61%
Single, divorced, widowed	9	39%
Education^**^		
College +	16	67%
Some college	7	29%
Trade school	1	4%
Employment status^**^		
Full time	9	38%
Part time	3	12%
Retired	12	50%
**High blood pressure**	22	88%
**Diabetes**	8	32%

* *Booster participants represent a sub-set of the Intervention group and are also included in counts for “Intervention”*.

** *Totals do not sum to 25 for some variables due to missingness/participant non-response*.

**Table 2:** Summary of key themes.

**Domain**	**Theme**	**Examples**
**Impacts**		
General impacts to daily life	Stay-at-home	Increased time spent at home; only leaving the house for essential service; fear of leaving the house
	Travel	Canceled trips for leisure, family events, and funerals; feelings of missed opportunity to travel during retirement
	Work	Loss of paid or volunteer work; transition to working from home
	Finances	Loss of income; feeling financially secure; concern about job security and the economy
	Policy impacts to behavior	Social distancing (maintaining 6 feet of space from others; keeping activities outdoors; minimizing contact when possible); masking in public; business closed or reduced access
Health and activity impacts	Mental health, energy, and stress	Experiencing low mood and symptoms of anxiety, elevated stress and concern for self, family, and friends; fatigued by ongoing stressors; lethargy; improved stress from lack of commuting
	Nutrition	Eating at home more often; getting takeout rather than dining at a restaurant; food cravings; weight gain
	Physical activity	More free time to be active; use exercise to cope and get out of the house; closed exercise facilities and canceled classes; not leaving the house means less daily movement; loss of social support for exercise; fear of leaving the house to exercise
	Sedentary time	Spending more time sitting because stuck at home; using tv and other seated hobbies to cope; replacing active errands with seated online orders
	Sleep	Difficulty sleeping due to stress; improved sleep due to work schedule changes
	Sickness/infection with COVID-19	Friends and family with infection; personal infection
Social impacts	In-person social connection	Fewer in-person interactions with family, friends, co-workers, and members of the community; limited in-person social circle; meeting outdoors and/or with masks
	Family events	Missed opportunities to see parents, children, and grandchildren; Missed milestone family events (funerals, graduations, the birth of babies, etc.)
**Coping strategies**		
Social connection	Virtual	Using phone, video chat, social media, and text to stay connected to friends and family; virtual connection less fulfilling; virtual connection provides unique opportunities for different engagement
	In-person	Limited outdoor gatherings with a limited network; reliance on spouse/partner in home for in-person connection
Activities	Hobbies	Watching TV; reading; crafts; online shopping; online social connection; online classes; gardening and yard or home improvement projects; engaging in racial justice and political activism
	Exercise	Opportunity to get out of the house; spending time outdoors walking or doing other activities; unstructured movement indoors to stay active; gave sense of normalcy; healthy activity they can still engage in
	Following public health guidance	Consciously making choices about behavior to follow public health guidelines, minimize risk of illness and/or ease anxiety; limiting trips in public; wearing a mask; avoiding places were other don't adhere to guidelines
	HART participation	Social connection with study staff; structure of goal setting and intervention schedule helpful; study content offers ideas for new coping strategies to try (e.g., meditation)
Beliefs	Positive attitude	Recognizing that they have faced and overcome other life hardships; taking things 1 day at a time; gratitude for their privilege; recognizing that others face greater hardship
	Spirituality	Belief that god has a plan; connection with others through religious services

### General Impacts to Daily Life

#### Staying at Home

As a result of the pandemic, most participants reported a large increase in the amount of time they spent at home. When they did leave their home, most reported being very intentional about their trips, limiting excursion to essential needs (groceries, banking, hardware purchases, etc.) and making those trips efficient to limit time spent away from home. For some essential services, like grocery shopping, many participants mentioned using delivery services or having others shop for them, at least some of the time. “…*I don't even want to go to the grocery store. I do every three weeks or something, but it's just that fear because I really don't want to get it*.” (P22). As a natural consequence of limiting time away from home, most participants reported a general decrease in social and physical activities due to the need to stay home and, often, feelings of increased isolation. “*Initially I was home all the time, I didn't go anywhere… It has been much more isolating for me*.” (P4). Many participants mentioned a distinct fear of leaving their home or going out, particularly in the early months of the pandemic. Leaving the house, even for essential reasons caused worry about exposure to the COVID-19 virus. Consequently, many equated staying home with staying safe. As time progressed and more information was available about the virus, many have adapted and began going out more in ways they deemed safe, typically engaging in mitigation measures like social distancing, mask use, and interacting in outdoor settings. “*And the more they started to learn about the virus and how you contracted…the more active I became*.” (P7).

#### Travel

COVID-19 disrupted many participants' travel plans for leisure, to visit family, or for specific events like a grandchild's high school graduation, reunions, or travel for funerals. Many participants found these disruptions upsetting and disappointing, viewing it as a missed opportunity. “*I didn't want to take this time out of my life to not travel and not see my family*.” (P25). One person delayed retirement because they would not be able to volunteer or travel, as planned. Despite this, some participants did report taking road trips, but generally limiting that travel to destinations closer to home and/or to second homes. “*I did go down to the Oregon coast for six days, so at that point where our beach home is, there wasn't so much issues with the pandemic*.” (P4).

#### Work

Work, whether paid or volunteer, was disrupted for many participants. For many, work typically provided a sense of purpose and encouraged them to get up each day, making these interruptions very disruptive to their daily lives. COVID ended most people's volunteer work, and several people reported losing some or all of their paid work. “*I was working. I lost my contract - I do contract work - due to COVID*.” (P18). Among those who were not retired and had continued employment, most transitioned to remote work-from-home arrangements. Responses to this transition were mixed among the portion of our sample that still worked full or part time. Some people appreciated the flexibility of working from home and appreciated the lack of commuting, while others reported finding it undesirable, less ergonomic, and challenging to incorporate into their home environment. “…*So working from home, I hate it, I absolutely hate it. My condo is not conducive to it*.” (P11). Many people also noted that in-person work had helped keep them active such as by taking stairs, visiting coworkers at their desks, or doing active work activities, all of which have ceased in a work-from-home environment. “*At work I've always tried to make a point of moving more, taking the stairs, things like that… But when I got home, and I don't hear anybody talking, it's completely quiet, I'm using equipment that's less ergonomically sensible yet I get so focused on my work that I just stay and do it and I don't move…*” (P19).

#### Finances

A few participants reported experiencing some level of financial hardship due to the pandemic. “*I just got told I'm being furloughed because the state has lost so much money, so I'm losing 20% of my salary*.” (P14). However, others felt financially secure or even able to save money. “*I've been able to save money. I'm not paying for gas, not paying for this and that…right now they're paying me my full salary, even though I don't work fulltime*.” (P11). Respondents also voiced uncertainty about their future job security, income, and the economy. “*I think the biggest impacts of COVID over time are going to be financial recovery*…” (P19).

#### Social Distancing, Masking, and Business Policy

Participants overwhelmingly reported adhering to social distancing recommendations and wearing masks outside their home. People reported intentionally minimizing in-person contact, maintaining space between themselves and others when engaging in in-person contact at work or in public, and keeping in-person contact focused on doing outdoor activities like walking or golfing. “…*[Golf]'s one thing I do all the time, 3-4 days a week, but we still social distance when we're playing. I try to make a point of trying to be six feet away*.” (P9). Reported masking habits differed between people and situations. Some wore masks all the time when not in the home, while others only wore them in populated public areas such as stores and parks, but not when walking around their neighborhood. “*I wear my mask and I keep my distance when I'm in public, but if I walk my dog [around the block] I don't wear a mask*.” (P5). A small portion of our sample voiced that they didn't like wearing them at all and avoided going places to avoid masking. Participants frequently voiced appreciation of people who wear masks in public, as well as a fear of and annoyance with people who don't. Most participants mentioned businesses they used regularly (e.g., pools, gyms, alternative medical providers, movie theaters) shutting down entirely. Furthermore, many participants mentioned that those businesses that remained open typically had rules for entry such as requiring masks, social distancing, temperature checking, or restricted hours of operation.

### Health and Activity Impacts

#### Mental Health, Energy, and Stress

Most participants voiced experiencing low mood, anxiety, stress, fear, and/or anger due to the pandemic. These feelings stemmed from high levels of uncertainty and major changes to their routines. These impacts were accentuated by the fact that activity restrictions meant more limited options for distraction. “*I have literally gotten depressed…As the months and weeks pass by, it's kind of like, what is going to happen? You just kind of wonder if it will end or if they will find a way to manage it better*.” (P7). Most participants indicated elevated and chronic stress from concern about their health, health of family and friends, finances, general disruption to daily life, as well as the divisive political climate. Furthermore, the ongoing nature of these stressors led to fatigue. “*I often feel more down because I don't see - none of us does - see an end to this style of living that we have to do now*.” (P1). A small number of participants reported lethargy and feelings of de-motivation with the pandemic. Interestingly, for a small portion of our sample, a shift to work-from-home and an end to commuting has meant a decrease in stress. “*My blood pressure has gone down, now that I work from home, which I find really interesting*.” (P11).

#### Nutrition

Eating habits were noticeably changed for most participants. Most people interviewed were not dining out at restaurants, though many reported still getting take-out to support local businesses. “*We stayed home and ate at home a lot. We have a favorite local restaurant that we had frequented, and they offered curbside pickup, so we took advantage of that…*” (P6). Most participants were eating at home more often, and grocery shopping habits have changed noticeably for many people. People reported shopping less frequently or ordering online, making it more challenging to keep fresh fruits and veggies easily accessible. “*I don't go out very often to shop for groceries so…it's harder to have fresh things all the time*.” (P12). Some participants noticed an increase in snacking between meals and more food cravings for less healthy food. “*I just felt a craving for, not so much sweets but dark chocolate. I've had different food cravings and not all of them healthy*.” (P5). Reports of weight gain as a consequence of stress, boredom, and diet changes were also common. “*Since March I've gained ten pounds, being inside. Even though I'm walking, I'm, out of boredom, looking for a ten o'clock snack and a two o'clock snack…*” (P8).

#### Physical Activity

Respondents reported large changes to their normal physical activity patterns. Some reported having more free time to engage in physical activity. Many reported being able to exercise the same amount as pre-pandemic or more, particularly through outdoor neighborhood walking. “*I started in January with a personal goal of ten thousand steps a day and so far I have averaged that every month…having the extra time to just go out whenever the weather looked was great versus having to wait until I was home from work*.” (P10). Several participants reported taking up home and yard improvement projects they might not have otherwise done. A small portion also reported doing virtual exercise classes or exercise videos (e.g., Zumba Gold, Silver Sneakers). Other participants reported that their physical activity routines were disrupted by the pandemic. Participants noted that the loss of normal activities and schedules generally meant moving less. Those going to gyms could no longer go. Many lost socially supported physical activities like walking groups or in-person classes. “…*I don't walk unless I have somebody to walk with because I just like that social interaction*…” (P4). Some reported they were too fearful of COVID to even leave the house to get outside. “…*For a little while I was afraid to leave, to go outside. I didn't know if you got it from the air…*” (P7).

#### Sedentary Time

Most participants noted a large increase in sitting time being stuck at home. “*Especially in the beginning…I felt like I was sort of a walking zombie. Well, sitting zombie*.” (P7). Many reported more TV watching and pursuing seated hobbies as an escape and coping mechanism. “*I watch television more than I ever have before, as an active escape mechanism*.” (P19). Activities like running errands were now done virtually while sitting on a computer rather than by leaving the house or walking around stores as they were before. “*So those early months I was seated a lot, looking at the computer trying to figure out how do I sign up for Instacart and how do I get the Costco app*…” (P7).

#### Sleep

Surprisingly, changes in sleep were not commonly reported by those interviewed. A few participants noted sleep disturbance and difficulty sleeping due to stress and worry about the pandemic. “…*under that kind of stress, you're not going to be sleeping well…it was kind of a vicious circle where you didn't sleep well, you're tired the rest of the day, all the problems start to magnify themselves because you're not rested enough to think clearly*.” (P7). However, a similarly small number noted that their sleep had improved due to changes in their work schedule or structure. “*I got better sleep and that was important, because before I was only getting maybe 5-6 hours and I work a 4/10 schedule, so I get better sleep now*.” (P2).

#### COVID-19 Infection

Several participants reported knowing a friend or family member who had COVID, including one who had an aunt who died from COVID-19. One participant reported personally having COVID-19 infection but had recovered at the time of the interview.

### Social Impacts

#### Changes to In-person Social Engagement

Because most participants were intentionally limiting visitors to their home and their own trips out of the house, they were not seeing friends, family, and people in the community as often as they did pre-pandemic. Many also mentioned specific loss of social engagement from prior regular activities such as exercise groups and classes, work and volunteer work, religious services, and community-facing errands. “*I couldn't do a lot of things that I've been doing for years. That was playing competitive badminton three times a week, I couldn't do that. I couldn't get up early and go volunteer in Seattle…*” (P2). This decrease in in-person social interaction resulted in reported feelings of social isolation and distress for many, though a small number of others reported not being bothered by it or even preferring limited social time. Some people did mention that they were still choosing to engage in in-person interactions with a limited network, albeit in different ways than normal (e.g., meeting outdoors, wearing masks, etc.). “*We have not had anyone in our home except our daughter, she's in our bubble of protection. She doesn't live with us. And we're very strict about masking and following the rules*.” (P24). Some participants specifically commented that, as older adults, they feel as if they are “losing time” to travel and see loved ones that they won't get back. “…*at my point in time in my life, assuming I have 20 more years left, I'm losing time. I didn't want to take this time out of my life to not travel and not see my family. So it's a little sad in that respect*.” (P25).

#### Family Events

Participants reported missing out on seeing parents, children, and grandchildren due to social distancing and travel resections including missing milestone family events like funerals, graduations, and the birth of new babies. “*I haven't been able to travel to see my grandchildren in D.C., there's a new baby coming and certain religious ceremonies that happen, and if it's a boy I can't be there*.” (P4).

### Coping Strategies

#### Social Connection

Because they were staying at home more and in-person social engagement is greatly reduced, many people reported transitioning social activities to virtual modes like phone calls, web-based video chat (Zoom, Facetime), email, web messaging (e.g., Facebook Messenger), and texting to help them cope and stay connected to others. “*We use Facebook and email and web so that keeps us in touch without having to be face to face, and that's worked fine for us*.” (P6). For many, the use of web-based video chats was a new skill, but many reported using it to attend religious services, group meetings (e.g., book clubs, bible study groups, choir practice, etc.), friend or family gatherings, happy hours, scholarly presentations, and online classes. “*We've tried to learn the new technology as much as possible so we can at least try to stay connected through different types of social media and using Zoom and stuff, to visit that way, and that's been really helpful*.” (P7). Participants noted that while these virtual media help them stay connected to friends and family, it is often not as fulfilling as in-person interaction. However, for some virtual connection was easier to fit into their lives than in-person meetings and has afforded unique opportunities to share experiences they wouldn't have otherwise (e.g., helping grandchildren with remote learning). Some participants noted that they thought their shift toward more virtual connection with friends and family may be a change that is carried forward beyond the pandemic, in place of some face-to-face visits. In addition to this large shift to virtual connection, some participants did report engaging in limited in-person connection in outdoor settings and with a limited group of individuals, as described in the In-person social engagement section above. Several participants also reported living with a partner who was supportive and provided a safe social connection. “*Well, I have an amazing husband, he seems to always be cheerful so it helps*.” (P25).

#### Hobbies

People reported engaging in activities and hobbies like watching television, doing crafts (e.g., knitting), and baking. “*I love mysteries, TV mysteries…That's my main coping mechanism. And of course shopping on Amazon*.” (P22). Many participants also reported spending time going online to browse, shop, connect with others, attend choir practice, or take classes (e.g., meditation and racial activism). “*But now we're listening a lot to online presentations by scholars and activists, especially on Black Lives and the shameful history of America*.” (P24). Many participants reported doing more gardening, tending to plants, or doing major yard and home improvement projects. “*I started to watch all these home improvement shows on TV, so I got these ideas and now I'm getting four new appliances next week and new flooring for my kitchen*.” (P2). Many also reported reading and one person engaged in an online book club. Several participants also mentioned becoming activists around politics and racial justice as means to work toward a more hopeful future.

#### Exercise

Physical activity, especially walking, was an important coping strategy for many participants, serving as a way for people to safely get out of the house, and do something that helped them stay healthy and spend time outdoors. Walking, home improvement projects and yard work were commonly reported ways to cope that had the additional benefit of allowing contact with nature and neighbors. “…*Getting out of the house and walking around the neighborhood, we did that every day, twice a day, and that gave you some sense of normality too*.” (P20). Because they were not getting as much daily life movement and many regular exercise options were not currently available, some participants also reported trying to get extra unstructured movement into their day to cope with the change. One person also started walking indoors in their home to get more daily steps.

#### Following Public Health Guidance and Minimizing Risk

Many participants acknowledged their high-risk status for COVID-19 infection and complications and noted fear about contracting the disease. To minimize risk of infection and help alleviate some anxiety and fear, many participants refrained from going out and preferred staying in to minimize risk of exposure to COVID-19. For many, this included trying to order groceries online, have someone else shop for them, or grocery shop infrequently. “*My community has organized people to shop for you, they'll come pick up my credit card and my store card and take my list and grocery shop and then bring it back to me*.” (P4). Additionally, when in public most participants noted intentionally social distancing and masking to minimize anxiety and infection risk. Many also reported avoiding places where people did not follow guidelines (such as areas of low mask usage. “*But at the park there's way too many people who don't believe in masks, so we just stick to the back streets in front of people's homes and stuff*.” (P8).

#### HART Participation

A few participants noted that being in the HART study was helpful during the pandemic, offering an opportunity for social connection with study staff, structure and goals, and ideas for new coping mechanisms to try. One person got the idea to start meditating during COVID-19 from conversations with their study health coach and study materials.

#### Beliefs and Attitude

Many participants reported coping by maintaining a positive, one-day-at-a-time attitude. Reflecting on past hardships and practicing gratitude for current privileges helped provide perspective and resilience coping with the pandemic. “*I am a person who takes it day by day, I don't get down. As hard as it is, there have been worse things that have happened in my life that caused me more anxiety and other issues than not seeing my grandkids. As hard as it is not having a regular routine. I have always had a positive outlook on life*.” (P4). For some, spiritual and religious beliefs and engaging in religious services and groups remotely were also helpful to maintain connection and perspective to cope with the challenges of COVID-19. “*I have a group of six gals, that we have been meeting online three times a week to talk and pray together. We're all Christians. Do a little Bible study. Just things to have some interaction. So that's been a good support group*.” (P10).

## Discussion

The COVID-19 pandemic and related public health mitigation measures have markedly changed nearly all facets of daily life and health for the older adults we interviewed. However, the resilience of this population was equally notable, particularly in their adaptability to new technologies and their ability to tap into past experiences to maintain a positive outlook despite hardships. The narratives shared by this sample provide great insight into opportunities for future intervention and research across areas of older adult health such as sedentary behavior, physical activity, mental health, and social engagement.

Almost universally, participants in our sample reported perceived increases in sedentary behavior since the onset of the pandemic due to the loss of routine and increased time spent at home and engaging in sedentary activities like watching TV. The older adult population is already known to be the most sedentary age group (18, 19), accumulating an average of 10–14 h of sitting each day, and consequently is at increased risk for numerous chronic health conditions (20–22). Indications that levels of sedentary time may be increased for many older adults during the indefinite pandemic period is concerning. Of note, much of that added sitting time was spent watching TV, which may be independently deleterious to health (23) and cognitive decline (24). However, future quantitative studies measuring the impact of these pandemic-related behavior changes on older adult sedentary behavior and health are warranted. Furthermore, these reported themes of increased sedentary behavior during this period of pandemic restrictions underscore the need for continued research into sedentary behavior interventions and health impacts for older adult populations, particularly those dealing with periods of isolation.

Interestingly, while some participants reported decreased physical activity as well as increased sedentary time, many participants reported doing more intentional physical activity. Activities like walking and gardening were commonly reported as coping strategies by our participants, as these activities helped them get outside the house, engage with neighbors and friends in safer ways, and maintain a sense of normalcy. Several participants also noted leveraging technology to engage in online exercise classes, an option that wasn't available pre-pandemic for most participants. This points to an opportunity for future research into optimal ways to incorporate virtual classes into older adult exercise programs, which could potentially expand program reach and maximize schedule flexibility for participants. Given the individual level variation in physical activity changes reported among older adults in our sample, future study to quantify the long-term impacts of the observed changes in physical activity is warranted.

Negative impacts on mental health and social engagement were also prominent in our sample. Participants expressed high levels of uncertainty and fear related to the pandemic and related governmental policies, leading to high levels of stress and worry. Further, the need to restrict socialization and limit travel was distressing for many participants, challenging many of their traditional coping mechanisms. Consequently, participants reported large shifts in the way they were staying socially engaged with friends and family, leveraging technology, particularly video conferencing, in ways they hadn't prior to the pandemic. Similar themes of stress, loneliness and coping through virtual social engagement are supported by two recent mixed methods studies conducted at during the onset of the pandemic, suggesting these themes are persisting over the course of the pandemic (9, 12). However, there is a need to ensure that older adults who are unfamiliar or uncomfortable with using technology are not left behind given that societally so many opportunities for social and physical activities have shifted to virtual delivery during this period, and likely beyond.

In general, the adaptability demonstrated in our sample to embrace new technological solutions and options to engage socially, exercise, and complete activities of daily living (like grocery shopping and banking) was notable. There are likely opportunities to leverage the new-found comfort in the older adult population with virtual connection. A 2017 Pew Research Institute report noted that internet use and comfort among seniors was steadily growing, with 67% of older adults age 65+ reporting regular internet use, including regular use of social media platforms among 34% of those age 65+ (25). Adding to this growing population-level comfort with and access to the internet, the increased knowledge and comfort with video conferencing platforms, like Zoom, precipitated be the pandemic could open up more and better opportunities for the public health, medical, research, and fitness communities to connect with older adults using virtual offerings (live video streams, recorded classes, etc.). Doing so could potentially expand the reach of these services to previously hard-to-reach sectors of the older adult community, such as those in rural areas and those with driving or other mobility limitations, and increase flexibility of services by allowing more on-demand offers that fit individual schedules. However, many populations of older adults, particularly those with lower educational attainment and socioeconomic status and those with self-reported physical disabilities, still face barriers to technological access and literacy and report less frequent engagement with technology (25, 26). Careful thought should be given to identifying and addressing barriers to technological literacy when developing these programs to avoid deepening existing disparities in access.

In addition to themes around technological adaptability, it was clear that positive attitude was a crucial coping mechanism among those interviewed. This manifested differently for various participants, with some adopting an attitude of persistence and others choosing to intentionally focus on gratitude for what they do have during this time of restriction. Drawing on the lifetime of prior stressors and challenges they had faced was a key source of resilience and strength that helped many to cope. Those participants that used this framework appeared to cope better with the frustrations and hardships of the COVID-19 restrictions, a phenomenon posited by Lind et al. (27), who suggest that older adults are better equipped to use past experiences to foster resilience and adaptability in times of stress. Another recent qualitative study of older adults in the US, also identified a positive attitude as a key coping mechanism for the hardships of the early phases of the pandemic and emphasized the resiliency of later life (28). This theme offers a lesson for all age groups and calls for more investigation into the use of mindfulness and gratitude training to build positive coping strategies for individuals of all ages experiencing chronic stress.

These findings must be interpreted in the context of several potential limitations. Like all qualitative studies, the findings may be unintentionally biased by the researchers. However, our team-based, iterative approach to analyzing and interpreting the data improved the rigor of our investigation. Social desirability bias may have resulted if participants felt compelled to describe their experiences during the HART study favorably. We attempted to reduce any perceived pressure by informing participants at the beginning of each interview that the study staff would not know the name of interview participants, that all feedback—even negative feedback—was valuable to the study team, and that participants were free to skip any question that they did not feel comfortable answering. Furthermore, participants were not offered additional incentives for completing the interview, and interviewers were neutral study team members who had no prior contact with interviewed participants. Generalizability of the results is limited by the use of a convenience sample of participants in the HART study. Participants who agreed to be interviewed may not be representative of the general population of older adults or of the larger HART study sample. Seasonality may also limit generalizability. We interviewed participants during the summer months (June–August), where outdoor recreation and socialization is more common and easier to facilitate. Due to the nature of our questions and pandemic-related activity restriction recommendations, perceived behavioral and health impacts of the pandemic may be experienced differently by participants in different seasons. Furthermore, our sample of older adults is predominantly white, highly educated, and all reside in and around the Seattle, WA area. Perspectives from this sample may not represent those of other older adult populations. Our sample also consisted of younger older adults (mean age = 68), who may be more tech savvy than the oldest old. Future studies should attempt to recruit a more diverse sample to see if qualitative experiences are shared across other populations.

In sum, the older adults in this sample noted impacts from the COVID-19 pandemic on nearly all facets of daily life and activity, particularly noting profound increases in sedentary time, stress, and social isolation. However, these older adults also demonstrated resilience and adaptability by embracing new technology and drawing on a wealth of life experience to cope with those changes. These coping strategies, particularly more extensive leveraging of technological interventions and mindfulness and gratitude training, can inform future research interventions for older adults dealing with chronic stress or isolation.

## Data Availability Statement

The raw data supporting the conclusions of this article will be made available by the authors, without undue reservation.

## Ethics Statement

The studies involving human participants were reviewed and approved by Kaiser Permanente Washington Institutional Review Board. The patients/participants provided their written informed consent to participate in this study.

## Author Contributions

MAGH, JD, and DR contributed to the study design, execution, coded transcripts, and conducted qualitative analysis. MAGH and JD carried out all primary data collection. JC, EH, JM, KM, and SP aided in data interpretation. All authors contributed to and approved the final manuscript.

## Conflict of Interest

The authors declare that the research was conducted in the absence of any commercial or financial relationships that could be construed as a potential conflict of interest.
