# Supplementary material for: “They're Going to Zoom It”: A Qualitative Investigation of Impacts and Coping Strategies During the COVID-19 Pandemic Among Older Adults
Source: Front Public Health. 2021 May 19;9:679976. doi: 10.3389/fpubh.2021.679976 (PMC8170019; doi:10.3389/fpubh.2021.679976)
Supplement: Supplementary file 1 [file Data_Sheet_1.docx]

**Supplemental Material**

**Appendix 1.** Codebook

**COVID Impact (I)**

What **impact** does the COVID-19 pandemic have on the daily lives of older adults?

Social (IS)

- Virtual (IS-V): changes in virtual social interaction habits
- In person (IS-IP): changes in in-person social interaction habits or social gathering habits (church, community events, etc.)
- Family events (IS-F): changes in participation in family or friend events (graduations, birthdays, funerals, births, in-person engagement with grandchildren or older parents/relatives)

Health (IH)

- Focus (IH-F): Changes to focus or concentration
- Energy (IH-E): Changes in amount of energy (more or less)
- Mental health (IH-MH): Changes in mental health or mood
- Nutrition (IH-N): Changes in diet, eating out habits, weight gain etc.
- Physical activity (IH-PA): Changes in physical activity levels
- Sedentary time (IH-ST): Changes in sedentary time and/or sedentary activities (e.g. TV watching, reading, crafts, etc.). Use in tandem with B-C (COVID-19 Barriers to sedentary behavior reduction) for I-STAND ppts.
- Sleep (IH-SL): Change in amount of sleep or sleep patterns
- Stress (IH-S): Increase or decrease in stress, anxiety, or fear
- Overall Health (IH-O): changes in overall feelings of wellness/health
- Sickness (IH-SK): of self, loved one, friends/neighbors
- Health care (IH-C): changes in how health care is accessed & administered
- Productivity (IH-P): changes in ability to accomplish everyday tasks
- Chronic conditions (IH-CC): changes in chronic health conditions like blood pressure

General Impact (IG)

- Future Plans (IG-P): Disruption of future plans (retirement, moving, etc.)
- Work (IG-W): changes in employment or volunteer work (work from home, loss of job, come out of retirement, work extra jobs, begin retirement, etc.)
- Travel (IG-T): changes in general travel plans, canceled trips, inability to plan new trips, etc. Both to visit family/friends, or for practical or leisure purposes. References to car trips or less/more traffic.
- Finances (IG-F): changes in personal finances and/or larger national economy
- Stay at Home (IG-SH): Reference not going out or leaving the home, self-quarantine, not going grocery shopping (at all or as much)
- No Impact (IG-N): No major changes or impacts due to COVID-19 identified

Policy (IP)

- Social distancing (IP-SD): Changes in behavior as a result of social distancing measures. Particularly mentions explicit actions of keeping space between themselves and others (e.g. 6 ft apart)
- Masks (IP-M): Changes in behavior as a result of mask/face covering policies
- Business policy (IP-B): changes in business or organizational policies that impact behavior as a result of appointment only retail or other services, excluding health care.
- Other (IP-O): changes in behavior as a result of policies not included above.

____________________________________________________________________________

**COVID Coping (C)**

How are older adults **coping** with the COVID-19 pandemic and associated stress, uncertainty and isolation?

Social (CS)

- Virtual connections (CS-VC): Regular phone calls/video chats or other non-in person connections (letter writing, texting, etc.) with family, friends, coworkers, etc.
- In-person connections (CS-PC): gatherings with friends and family (socially distant/outside or not) or obtaining services (haircut, massage, nails, etc.)

Activities (CA)

- Hobbies (CA-H): Engaging in hobbies, passive activities/escapism (TV, reading, crafts, gardening, etc.), or any activity used to stay busy as a coping mechanism (could include work, taking a class)
- Home Improvement (CA-HI): Focusing on home/yard projects, rearranging living space or changing living space to better suit changes to personal situation
- Exercise (CA-E): Engaging in exercise
- Outdoors (CA-O): spending time in nature
- Guidance (CA-G): Following/adhering to CDC and local guidance (wearing mask, washing hands/hand sanitizer, social distance, staying in when possible, not travelling, etc.)
- HART Participation (CA-P): Participating in the HART study

Beliefs (CB)

- Attitude (CB-AT): Positive attitude, taking it day by day, feeling fortunate, counting blessings, happy with where they are, enjoy minimizing social interaction, staying relaxed
- Activism (CB-AC): Political involvement (donating, protesting, calling/letter writing, etc.); acts of service for the community (volunteering, donating money, blood, medical supplies)
- Spiritual (CB-S): involvement in religion/spirituality
- Risk Minimizing (CB-R): staying indoors, avoiding certain activities, reducing trips out, etc. with the belief that this will minimize exposure risk

**Appendix 2.** Additional supporting quotes illustrating impacts from the COVID-19 pandemic to daily life and health and the strategies used to cope with these impacts.

| **General Impacts to Daily Life** | |
| --- | --- |
| Stay-at-home | Initially I was home all the time, I didn't go anywhere… It has been much more isolating for me. – P4  …I don't even want to go to the grocery store. I do every three weeks or something, but it's just that fear because I really don't want to get it. – P22  We don't go out unless we absolutely have to. I have all my supplies delivered to my house. I don't go to the grocery stores. – P7  But it's still a concern because we feel like we're shut in. We're the population that isn't really allowed to go out because you just don't know how well the young people are protecting themselves or others. You want to trust everybody, but at the same time it's one of those things that you're foolish if you do.” – P7  I don't go anywhere. I'm afraid of it. I have a lady friend and she's also afraid of it, so we talk on the phone. I've been over to her house a couple times, but we don't go anywhere because of this virus. … I'd like to do things, go out more, but obviously I can't. – P21 |
| Travel | My fiftieth high school reunion didn't happen in September in New England, our trip to Scotland in September didn't happen. But again, as a privileged white person, these are minor things that can be rescheduled at some other time. – P15  I could retire next year, I could retire anytime - but I can't travel or do anything, like my traveling and volunteering, so why retire? – P2  I think the ability to travel is going to be a long time before you can do that. It's moderately frustrating because my son lives in Hawaii and I was planning to go see him and now we can't. – P12  I didn't want to take this time out of my life to not travel and not see my family. So it's a little sad in that respect. – P25  Plus we haven't had a vacation, we can't go anywhere. Keeping in touch with my family in the UK has been challenging. We just haven't been anywhere. – P14  I did go down to the Oregon coast for six days, so at that point where our beach home is, there wasn't so much issues with the pandemic. – P4  We're about to head off on this monster trip to go do all that, for a month, driving, camping, hiking. This Utah-Colorado deal. A dozen mountains and a hundred some miles, God knows how many miles driving, but anyhow it's all specked out. It's possible to do camping and hiking - not in a campground, dirt camping. – P15 |
| Work | At work I've always tried to make a point of moving more, taking the stairs, things like that…But when I got home, and I don't hear anybody talking, it's completely quiet, I'm using equipment that's less ergonomically sensible yet I get so focused on my work that I just stay and do it and I don't move here the way that I would – oh, I need to call this person, instead of calling this person I'll just go to that person's desk. Well, that's not an option anymore. – P19  I couldn't get up early and go volunteer in Seattle, that would make me get up and go. – P2  In the beginning some people were going to be allowed to go up to do certain types of work, but because I was over 65, I was not allowed at all. So working from home, I hate it, I absolutely hate it. My condo is not conducive to it. – P11  But I'm in a situation where I can do my work from home. Pretty much everything I do in the office I can do at home… I probably will continue to work at home, even when a lot of other people go back to their offices. – P13  I was working. I lost my contract - I do contract work - due to COVID, but the money doesn't really affect my lifestyle, I don't need it that much, but it is a purpose and something to do if you're working. – P18  For me, it means I've had to work at home which is great, because I do not have to commute 62 miles to work and back every day…Negative side - I just got told I'm being furloughed because the state has lost so much money, so I'm losing 20% of my salary. – P14 |
| Finances | We're privileged and lucky to be financially stable and no longer employed, not having to figure out like most adults a) unemployment, b) working from home. We don't have dependent children we're raising anymore, having to figure that out. – P24  I've been able to save money. I'm not paying for gas, not paying for this and that. – P11  …right now they're paying me my full salary, even though I don't work fulltime. I know behind the scenes that's been a bit of an issue. So I wonder how long that's going to continue. That's really going to hurt, because even though I'm old enough to retire, I can't afford to retire right now. Once that comes to a head, that's obviously going to bring up the stress again. – P11  I just got told I'm being furloughed because the state has lost so much money, so I'm losing 20% of my salary – P14  I think the biggest impacts of COVID over time are going to be financial recovery – P19  And this pandemic has just proven the failure of our government, of our economic system – P24 |
| **Policy-Related Impacts to Behavior** | |
| Social Distancing | I have walked with friends, distanced and wearing a mask, but that's been rare. – P4  I wear my mask and I keep my distance when I'm in public. – P5  With that in mind, down here they allow golfing, that never stopped. So that's one thing I do all the time, 3-4 days a week, but we still social distance when we're playing. I try to make a point of trying to be six feet away. – P9  We're pretty serious about the social distancing and masks and all that. I'm kind of amazed at the fact that people don't do that. - P18 |
| Wearing Masks | I can go out and about and all that, but to get out of the building I have to mask up, I can't just pop out and pop back in. – P3  I wear my mask and I keep my distance when I'm in public, but if I walk my dog [around the block] I don't wear a mask. – P5  Wearing a mask - the very few times when I have gone out, it's a real pain, I don't like it. I do it, of course, but it's a real pain. – P1  “If I see anybody coming that isn't masked, we turn around and go the other way. But I'm really impressed with the number of people in my neighborhood that are masked. P7  Going to stores we always wear the mask. We think it's a good thing. We think it's a shame to see people not wearing masks in stores and even people who have them around their head or their neck - why are you wasting your time when you don't wear it? My thought is why are people so selfish?” – P9  “The park… is just no fun anymore. We used to do that almost daily, 2-3 circles around the park. But at the park there's way too many people who don't believe in masks, so we just stick to the back streets in front of people's homes and stuff. – P8  We're very strict about masking and following the rules. – P24 |
| Business Policy | The gym thing is a bigger challenge, I think. I was starting to go there, starting to plan to do some working out, and it all shut down. – P13  My best friend lives at… a high-end retirement home and it's been closed to the public for seven months. I've not seen my best friend. She was in the hospital. I couldn't go to the hospital to see her. – P22 |
| **Health & Activity Impacts** | |
| Mental Health, Energy, & Stress | Everybody's scared stiff. I'm sick of hearing about how many people can't get a job and can't pay their rent, everybody's going bankrupt. It's kind of hard to maintain perspective that there's hope out there. – P22  I often feel more down because I don't see - none of us does - see an end to this style of living that we have to do now. – P1  So between the worry, the do nothing, the sitting around, the inability to really focus or think, it ended up being a big puddle of nothingness, except for gloom and doom and worry and what do we do, for several months. – P7  Initially, when this all happened, it was quite depressing. I would sit on my couch and just flip the channels, watch TV for hours, and I would eat more, and I can't exercise or nothing. I wasn't doing good then. – P2  I really think the toll on mental health is far worse than anything. … Everything makes me mad, and I don't usually get mad. I guess it's better than being depressed. – P23  I just lose track of time. Like I said, I wind up sitting around in my pajamas until four o'clock on some days and then I think - why put clothes on? I might as well switch to my afternoon pajamas. I lose track of time, I can't get anything done. And I'm not meeting any goals. – P23  A feeling of just totally lethargic, and where do we go from here, just sit and stare at the TV. To the point where I couldn't even focus on a book. It took too much energy to even concentrate on a book… - P7  I mean, you worry about family or friends that could be in trouble, whether it's financial or health. – P6  …if either one of us got this, we would probably both die because of our health. And that’s really scary. – P1  So I don't know where we're headed. It's an enormous thing. 9/11 was huge, the Great Recession was huge - this is far bigger. – P15 |
| Nutrition | We stayed home and ate at home a lot. We have a favorite local restaurant that we had frequented, and they offered curbside pickup, so we took advantage of that… - P6  I've gained a few [pounds] back here recently, but I think that's been out of frustration. It was something of a side effect of the pandemic. – P5  I just felt a craving for, not so much sweets but dark chocolate. I've had different food cravings and not all of them healthy. – P5  But as far as my health? Well, because of COVID, it got a little bit worse. I have to get back to more exercise, better healthy eating, drinking more water. I already can get good sleep, which is good, but I need to lose the 7 pounds I gained back. – P2  Since March I've gained ten pounds, being inside. Even though I'm walking, I'm out of boredom looking for a ten o'clock snack and a two o'clock snack, where otherwise I'd be out walking or going to class or that kind of thing. – P8  I don't go out very often to shop for groceries so you end up using more things - it's harder to have fresh things all the time. I got stuck in a rut with carrots and things that would last. Because I used to shop a couple times a week so I could always have fresh fruit, fresh vegetables, that sort of thing. – P12 |
| Physical Activity (PA) | ***Less PA***  I'm not by nature an athletic person, I don't walk unless I have somebody to walk with because I just like that social interaction. – P4  But I don't get out as much for exercise. Even just going for a walk, I don't do… - P1  Like taking the dog out for a walk. For a little while I was afraid to leave, to go outside. I didn't know if you got it from the air. I just didn't know…But now things are better. I feel more confident. I go for walks… - P7  I couldn't do a lot of things that I've been doing for years. That was playing competitive badminton three times a week, I couldn't do that. – P2  So twice a week Zumba at the 24-Hour Fitness and then twice a week of the Silver Sneakers. So Golden Zumba and Silver Sneakers. That was 4 days a week I cut back right there. So I've had to do a lot more of dance in front of the TV. I make myself stand up to watch the news and do my dancing while I watch the news…And then 2-3 times a week we go outdoors and walk in our streets of our neighborhood….” - P8  I don't go out. I do go out in the yard, but I don't really go out and walk around in the street, because you see everybody in masks and then it's like oh God, this really is a dystopian disaster. Where if I stay in the house, or around my house, I at least don't have visual reminders of everything. – P23  The biggest thing is I can't go to the gym, but I am substituting video workouts. - P17  ***More PA***  And like I said, my husband and I have been doing a lot of gardening. We just decided to tackle a total garden makeover we originally wanted professional people to do, but I looked at it and said we're going to start this. We're going to do some of this ourselves, and we have. - P10  I started in January with a personal goal of ten thousand steps a day and so far I have averaged that every month…That's been a positive impact and having the extra time to just go out whenever the weather looked was great versus having to wait until I was home from work. - P10  Going out, getting out of the house and walking around the neighborhood, we did that every day, twice a day, and that gave you some sense of normality too. – P20 |
| Sedentary Time | I watch television more than I ever have before, as an active escape mechanism. - P19  Especially in the beginning, … I just stayed in because they didn't really know how you caught the virus… So I didn't really go outside. … When I'm in the house, I do sit a lot. I sew and do other things, but for a little while I felt like I was sort of a walking zombie. Well, sitting zombie. – P7  Initially, when this all happened, it was quite depressing. I would sit on my couch and just flip the channels, watch TV for hours, and I would eat more, and I can't exercise or nothing. – P2  I don't get up as much. I never realized how often I got up at the office…How often I would get up to go to the printer. How often I would get up to sort the mail and then distribute it. - P11  I'm just sitting here in the house. I can't see anybody, I can't do anything. – P23  Sitting time was already a challenge, because working at home at a computer job I'm sitting a lot anyway. I've tried to be getting up and moving around a little bit during the day and then go out for walks in the afternoon. So I'm looking forward to things opening up more, now that the parks are open. - P13  So those early months I was seated a lot, looking at the computer trying to figure out how do I sign up for Instacart and how do I get the Costco app. There was a lot of learning I had to do to figure out how can I manage this and not go out. – P7 |
| Sleep | I got better sleep and that was important, because before I was only getting maybe 5-6 hours and I work a 4/10 schedule, so I get better sleep now. – P2  It became really ridiculous there for a little while. And then, of course, under that kind of stress, you're not going to be sleeping well or anything, and so it was kind of a vicious circle where you didn't sleep well, you're tired the rest of the day, all the problems start to magnify themselves because you're not rested enough to think clearly. – P7 |
| Sickness/Infection with COVID-19 | But I got a call and we're in a condominium building of 60 units, and as it turned out, 5 people in our building of 60 units had the virus. - P8  I was being very good about staying home and not getting exposed to anyone, but I think I either got it from my husband or my doctor. I don't know who. – P20 |
| **Social Impacts** | |
| Changes to In-Person Social Connection | I couldn't do a lot of things that I've been doing for years. That was playing competitive badminton three times a week, I couldn't do that. I couldn't get up early and go volunteer in Seattle… - P2  Of course our social life has declined, just getting out and exercising hasn't been quite as easy. I usually do that with friends and some of that's been changed. And it's depressing. -P3  We have some select friends that are about our same age, they're the only ones we have ever gotten close to physically. We know their history, we know what's going on, so we're very selective about who we interact with. -P6  My kids don't visit me, and rightfully so. I totally understand where they're coming from. I haven't seen [my daughter] in forever and I miss her and I want to go to her house because she just bought a new home in January and she wanted to do her garden and all that, and I can't help. -P7  I still communicate with family and friends by phone, but it's not the same because you can't go to family parties and events and stuff. I've just got to deal with it, I think. I don't know how I dealt with it. I just deal with it. – P2  We have not had anyone in our home except our daughter, she's in our bubble of protection. She doesn't live with us. And we're very strict about masking and following the rules. – 10842  I've seen face to face friends five times since the beginning of March and most of them were very short visits, just dropping things off - open up the trunk, I step away, they get it. About two weeks ago I did visit with a friend on her front yard. We were 10 feet away and it was a lifesaver, it was so important. I really needed that. But it's been really hard, especially not seeing any end in sight. - P11  The thing I think about most is that at my point in time in my life, assuming I have 20 more years left, I'm losing time. I didn't want to take this time out of my life to not travel and not see my family. So it's a little sad in that respect. – P25  Not being able to hug my grandchildren, and be in person with them, except with a mask at distance. That's my number one thing. Not being able to go to church on Sunday with the people I dearly love, and sing and all of that. - P15  I can work fulltime, get paid fulltime, and never leave my house. I'm not very social, so one of the hard things for me in life is human interaction, and now I don't have to. – P19 |
| Family Events | I haven't been able to travel to see my grandchildren in D.C., there's a new baby coming and certain religious ceremonies that happen, and if it's a boy I can't be there. – P4  As far as our personal lives, the biggest impact was we were planning to spend some time with our grandchildren in California this summer. In fact my oldest granddaughter graduated from high school this year so that was all messed up. We didn't travel. It was pretty upsetting for a while, more so for my wife than me. – P6  I miss going out and shopping for my little nieces and nephews' birthdays. I miss not being able to visit the small kids whenever there is a birthday or a holiday. It has been really difficult. – P7 |
| **Social Connection to Cope** | |
| Virtual Interactions | We use Facebook and email and web so that keeps us in touch without having to be face to face, and that's worked fine for us. We were doing that before the pandemic. We might be using it more as an alternative to face to face. – P6  I have two friends I talk to for a half an hour each on the phone every week. We listen to each other for a half hour each so that's good. It's not the same as being able to sit in a nice little café. – P22  But at the same time we've tried to learn the new technology as much as possible so we can at least try to stay connected through different types of social media and using Zoom and stuff, to visit that way, and that's been really helpful. – P7  I talk to my daughter more on the phone than I ever have before, and that's kind of nice. – P7  I still communicate with family and friends by phone, but it's not the same because you can't go to family parties and events and stuff. I've just got to deal with it, I think. – P2  We're doing Zoom meetings, which luckily we have a couple gals in our book group who are hand at doing that, so every month we've had our book group meeting on Zoom. We just sit at our dining table and have our own cup of coffee by ourselves with the meeting. That's been a thing that's certainly helped. What else? We did a Zoom cocktail hour one Friday with our exercise group, our 24 fitness group. – P8  I have a group of six gals, that we have been meeting online three times a week to talk and pray together. We're all Christians. Do a little Bible study. Just things to have some interaction. So that's been a good support group. -P10  I think all of us have discovered Zoom and we've taken some classes. But now we're listening a lot to online presentations by scholars and activists. – P24  I've been reading to my grandson five days a week, who's in Alaska so we've been Facetiming every day. Which has been really actually good because he hasn't been in school so as part of his activity, I've been reading chapter books with him. That's been a good thing. That's one thing we've done to help. His mom works so she's able to do stuff while he's on the phone, and then Grandpa does math with him later in the afternoon – P3 |
| In-Person Interactions | I have walked with friends, distanced and wearing a mask, but that's been rare. Sometimes I've had friends come over, they'll sit on my rockery and I'll sit on my front porch area and we'll just sit and talk so that's been nice. We sometimes have had a picnic that way too… It's been isolating a lot… - P4  But it is really nice there are two of us who are here most of the time, so we're not totally isolated either - P1  About two weeks ago I did visit with a friend on her front yard. We were 10 feet away and it was a lifesaver, it was so important. I really needed that. - P11 |
| **Activities to Cope** | |
| Hobbies | I love mysteries, TV mysteries. They have the international mystery channel. That's my main coping mechanism. And of course shopping on Amazon. – P22  I watch television more than I ever have before, as an active escape mechanism. - P19  I started to watch all these home improvement shows on TV, so I got these ideas and now I'm getting four new appliances next week and new flooring for my kitchen. – P2  We're doing Zoom meetings, which luckily we have a couple gals in our book group who are hand at doing that, so every month we've had our book group meeting on Zoom. We just sit at our dining table and have our own cup of coffee by ourselves with the meeting. That's been a thing that's certainly helped. - P8  I have been doing a lot of gardening. We just decided to tackle a total garden makeover we originally wanted professional people to do, but I looked at it and said we're going to start this. We're going to do some of this ourselves, and we have. So that's been good. - P10  But now we're listening a lot to online presentations by scholars and activists, especially on Black Lives and the shameful history of America. - P24  I've read all these stupid books…Usually I would read like four of those kind of books in a year. I've read like two whole garbage bags full of them, and there just basically is nothing to do. – P23  Well, we have been politically involved and it's confirmed that. The last month with the Black Lives Matter movement, we're very engaged with that. Actually having that come to the forefront is something that is hopeful, that it's come to the forefront of so many white people in new ways. – P24 |
| Exercise | I have walked with friends, distanced and wearing a mask, but that's been rare. – P4  My purpose is now to walk the dog more often. - P18  Going out, getting out of the house and walking around the neighborhood, we did that every day, twice a day, and that gave you some sense of normality too. – P20  Regular walking around Green Lake a hundred something times during COVID -19 hasn't killed me, but to not walk around Green Lake a hundred times could. - P15  But I have my smart watch and I do my walking indoors. – P8 |
| Following Public Health Guidance & Minimizing Risk | ***Following Public Health Guidance***  I think the state government's been good. I know a lot of people claim it's infringing on their freedoms, but so is wearing a seatbelt. – P6  …we just take one day at a time and follow as many safety protocols as we can. – P7  But at the park there's way too many people who don't believe in masks, so we just stick to the back streets in front of people's homes and stuff. - P8  We have driven a couple times up to the Bellevue Botanical park and that's very nice and they have a note outside, "please wear masks" and everyone's been wearing masks there. So that's a nice place. - P8  ***Risk Minimizing***  My community has organized people to shop for you, they'll come pick up my credit card and my store card and take my list and grocery shop and then bring it back to me. In my community I have not been grocery shopping. – P4  I don't even want to go to the grocery store. I do every three weeks or something, but it's just that fear because I really don't want to get it. – P22 |
| HART Participation | In terms of when COVID started, meeting on the phone was great so that was really helpful. I appreciated that. – P4  One of the things before the surgery that I had set as a goal with Erica was trying out meditation, which I've never really done in an organized way. I was doing it on a very kind of low level 15 minutes a day. I wouldn't have done that. except that was an idea that came to me through the study. - P10 |
| **Beliefs to Cope** | |
| Positive Attitude | I am a person who takes it day by day, I don't get down. As hard as it is, there have been worse things that have happened in my life that caused me more anxiety and other issues than not seeing my grandkids. As hard as it is not having a regular routine. I have always had a positive outlook on life. – P4  There's a lot of people with a lot more difficulties than we're having so we were fortunate. – P6  It's kind of hard to maintain perspective that there's hope out there. I have friends in this building who lived in London during the war when it was bombed for how many months. So I try to keep a perspective there, maybe it's not quite as bad as that. – P22  I hate to say this because it's such a horrible thing for so many people in so many ways, but it actually works out really well for me. I can work fulltime, get paid fulltime, and never leave my house. I'm not very social, so one of the hard things for me in life is human interaction, and now I don't have to. - P19  What we do in my household, we just take one day at a time and follow as many safety protocols as we can. -P7  We're privileged and lucky to be financially stable and no longer employed, not having to figure out like most adults a) unemployment, b) working from home. We don't have dependent children we're raising anymore, having to figure that out. We have excellent health going in, even though we're at a supposed at-risk age group. We have excellent access to health care if we need it and a really comfortable home to live in. We could sit out on the porch and have breakfast and dinner every day, it's a lovely environment. It's not the cataclysmic change in our lives that so many people are dealing with. -P24 |
| Spirituality | God knew a long time ago this was going to happen, and He helped the friends [at our church] to prepare for it so when it came, we had a path. – P13  I have a group of six gals, that we have been meeting online three times a week to talk and pray together. We're all Christians. Do a little Bible study. Just things to have some interaction. So that's been a good support group. – P10  So the church does video services every week and I'm involved in those. I do the assistant minister job, or I do the job of the person who reads all the readings and things…It actually becomes quite a bit of contact with other people. – P12 |
